# A 3D human co-culture to model neuron-astrocyte interactions in tauopathies

**DOI:** 10.1186/s12575-023-00194-2

**Published:** 2023-02-23

**Authors:** Kevin L. Batenburg, Claudia Sestito, Paulien Cornelissen-Steijger, Jan R. T. van Weering, Leo S. Price, Vivi M. Heine, Wiep Scheper

**Affiliations:** 1 Department of Functional Genomics, Center for Neurogenomics and Cognitive Research, Vrije Universiteit Amsterdam, Amsterdam Neuroscience - Neurodegeneration, De Boelelaan 1085, 1081 HV Amsterdam, The Netherlands; 2 Crown Bioscience Netherlands B.V. (Formerly OcellO B.V.), Leiden, The Netherlands; 3 Amsterdam UMC location Vrije Universiteit Amsterdam, Department of Child and Adolescent Psychiatry, Amsterdam Neuroscience, De Boelelaan 1085, 1081 HV Amsterdam, The Netherlands; 4 Department of Complex Trait Genetics, Center for Neurogenomics and Cognitive Research, Vrije Universiteit Amsterdam, Amsterdam Neuroscience, De Boelelaan 1085, 1081 HV Amsterdam, The Netherlands; 5 Amsterdam UMC location Vrije Universiteit Amsterdam, Department of Human Genetics, Amsterdam Neuroscience - Neurodegeneration, De Boelelaan 1085, 1081 HV Amsterdam, The Netherlands

**Keywords:** hiPSC-derived neurons, Astrocytes, Neuron-astrocyte interaction, 3D co-culture, Tau aggregation

## Abstract

**Background:**

Intraneuronal tau aggregation is the major pathological hallmark of neurodegenerative tauopathies. It is now generally acknowledged that tau aggregation also affects astrocytes in a cell non-autonomous manner. However, mechanisms involved are unclear, partly because of the lack of models that reflect the situation in the human tauopathy brain. To accurately model neuron-astrocyte interaction in tauopathies, there is a need for a model that contains both human neurons and human astrocytes, intraneuronal tau pathology and mimics the three-dimensional architecture of the brain.

**Results:**

Here we established a novel 100–200 µm thick 3D human neuron/astrocyte co-culture model of tau pathology, comprising homogenous populations of hiPSC-derived neurons and primary human astrocytes in microwell format. Using confocal, electron and live microscopy, we validate the procedures by showing that neurons in the 3D co-culture form pre- and postsynapses and display spontaneous calcium transients within 4 weeks. Astrocytes in the 3D co-culture display bipolar and stellate morphologies with extensive processes that ensheath neuronal somas, spatially align with axons and dendrites and can be found perisynaptically. The complex morphology of astrocytes and the interaction with neurons in the 3D co-culture mirrors that in the human brain, indicating the model’s potential to study physiological and pathological neuron-astrocyte interaction in vitro*.* Finally, we successfully implemented a methodology to introduce seed-independent intraneuronal tau aggregation in the 3D co-culture, enabling study of neuron-astrocyte interaction in early tau pathogenesis.

**Conclusions:**

Altogether, these data provide proof-of-concept for the utility of this rapid, miniaturized, and standardized 3D model for cell type-specific manipulations, such as the intraneuronal pathology that is associated with neurodegenerative disorders.

**Supplementary Information:**

The online version contains supplementary material available at 10.1186/s12575-023-00194-2.

## Background

Tauopathies are a class of neurodegenerative diseases that are characterized by the progressive aggregation of tau protein and include Alzheimer’s Disease (AD) and frontotemporal dementias (FTD) such as Pick’s Disease, Corticobasal Degeneration and Progressive Supranuclear Palsy. Since tau is abundantly expressed in neurons where it predominantly accumulates in disease conditions, it disrupts neuronal physiology and leads to neurodegeneration in a cell-autonomous manner. In addition, astrocytes are affected by neuronal tau pathology and actively contribute to neurodegeneration by the loss of homeostatic function and gain of toxic function [1–3]. Rodent models have generated important insight into how cell (non-) autonomous disease pathways converge and eventually lead to neurodegeneration [3–5]. However, mouse models are relatively expensive and often only recapitulate limited aspects of human disease [6]. More importantly, many therapeutic targets derived from rodent studies have thus far failed in clinical trials, emphasizing the need to establish human models of tau aggregation.

Human induced pluripotent stem cell (hiPSC) technology has created the possibility to study disease mechanisms in human neurons and astrocytes. The physical and functional interaction between these cell types, for example at tripartite synapses, is critical for brain physiology and plays an important, yet not fully understood role in neurodegeneration [7]. Recently our group developed a two-dimensional (2D) hiPSC-derived neuron/astrocyte co-culture model for tau pathology, to study cell non-autonomous effects of intraneuronal tau pathology [8]. Although hiPSC-derived neural cultures contributed to our understanding of neuron and astrocyte physiology, they lack the natural interactions in the brain micro-environment. This is a limitation for astrocytes to acquire their in vivo*-*like morphology and thus the complexity by which they interact with neurons in a three-dimensional (3D) network [9, 10]. This led to the development of 3D neuron/astrocyte models that display increased cellular and spatial complexity [11, 12], but these systems typically depend on the self-organization into organoids that are complex, time-consuming, costly and may suffer from heterogeneity and limited throughput. Organoids are valuable assets to investigate neurodevelopmental disorders [13], but are thus far less well suited for the study of aging-associated neurodegenerative disease. Moreover, the robust induction of intraneuronal tau pathology has not been reported in 3D organoids.

Therefore, the aim of this study is to establish a physiologically relevant and robust model representing 3D neuron-astrocyte interactions. To this end, we present a step-by-step procedure to generate 100–200 µm-thick 3D microwell co-cultures of hiPSC-derived neurons and human primary astrocytes. This system is miniaturized, compatible with cell type-specific manipulation as well as standard imaging techniques, and can be efficiently generated at reduced cost. We show that neurons in the 3D co-culture form a morphologically mature synapse pattern and display spontaneous calcium activity within 4 weeks. Moreover, like in the human brain, astrocytes display a complex morphology with extensive processes that enwrap neuronal somas, extend upon axons and dendrites, and localize perisynaptically. As a proof-of-concept for the cell type-specific introduction of pathology in neurons, we successfully established seed-independent intraneuronal tau pathology in the 3D co-culture to enable the study of the role of neuron-astrocyte interactions early in the pathogenesis of tauopathies. Altogether, we established a rapid and robust miniaturized model that mimics the 3D context of the human brain to study the interaction between neurons and astrocytes and how this is altered in tauopathies.

## Results

### Development of a 3D human neuron/astrocyte co-culture

**Fig. 1:**
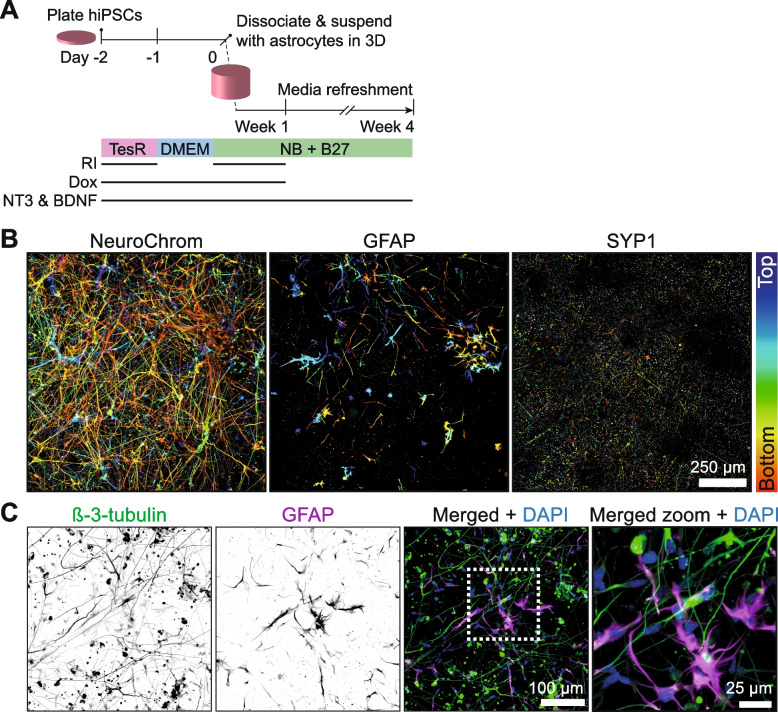
Development of a 3D human neuron/astrocyte co-culture. **A** Schematic representation of the protocol to generate the 3D human neuron/astrocyte co-culture. Ngn2-hiPSCs are plated on day -2 for neural induction in stem cell (TeSR) medium containing ROCK inhibitor (RI), doxycyclin (dox), NT3 and BDNF. One day after medium (DMEM) change on day -1, Ngn2-neural progenitors are detached on day 0 and mixed with human primary astrocytes in medium and Geltrex for neuronal maturation in medium (NB + B27) containing NT3 and BDNF with weekly media refreshments for 4 weeks. **B** 100 µm-thick maximum intensity confocal image projection of neurons and astrocytes in 3D co-culture. Immunostaining was performed for neurons (NeuroChrom), astrocytes (GFAP) and presynapses (SYP1). Single channels are shown as a heatmap corresponding to the focal plane of the fluorescence (bottom, red; top, blue). **C** 160 µm-thick maximum intensity confocal image projection of neurons and astrocytes in 3D co-culture. Immunostaining was performed for neurons (β-3-tubulin, green) and astrocytes (GFAP, magenta). Single channels are shown in greyscale and the merged image includes nuclei (DAPI, blue). A zoom of the boxed area indicated in the merged image is shown

We developed a protocol for the rapid generation of miniaturized and homogeneous 3D neuron/astrocyte co-cultures (Supplementary Document 1). Briefly, we employed a clonal hiPSC-line with doxycyclin-inducible Neurogenin 2 (Ngn2) [14], that was previously shown to facilitate the efficient generation of glutamatergic neurons from hiPSCs [15, 16]. Ngn2-hiPSCs were pre-differentiated to neural progenitors for two days prior to 3D co-culturing with human primary astrocytes. This time-window of separate neuron/astrocyte culturing enables cell type-specific manipulations such as viral transduction. Next, neural progenitors were detached and mixed with human primary astrocytes in medium mixed with Geltrex extracellular matrix (ECM) and supplemented with NT3 and BDNF for neuronal maturation for 4 weeks (Fig. 1A). Since we observed proliferation of astrocytes in the 3D culture, the neuron/astrocyte ratio was optimized to prevent overgrowth and subsequent clumping of astrocytes. To this end, 30.000 neural progenitors and 5.000 human astrocytes were mixed per well: a cell ratio and density that resulted in a homogeneous distribution in the 3D co-culture, thus facilitating subsequent microscopy analyses. Also, we observed that a cell suspension with 50% Geltrex (v/v), but not lower concentrations, polymerized into sufficiently thick 3D co-cultures that remained stable for at least 4 weeks. Finally, miniaturization of the protocol enabled the maintenance of 50 µL 3D co-cultures that are shaped as a thin cylinder on the bottom of 96-well microplates. In this format the co-cultures are easy to handle and can be maintained by media refreshment once every week. In addition, since the microwell plates with a high-clarity foil bottom are compatible with imaging, this enabled the immediate processing of the 3D co-cultures within the plate. Altogether, these characteristics of our 3D neuron/astrocyte platform are assets to increase the throughput and reduce handling and costs.

To investigate neuronal differentiation in 3D co-culture with human astrocytes, we performed immunofluorescent labeling using a combination of neuronal and astrocytic markers after 4 weeks of 3D co-culture. These data show that the 3D ECM permits the diffusion of antibodies at similar concentrations used for 2D cultures and confirm the compatibility of the 3D system with confocal microscopy. Confocal imaging within the 100 μm-thick 3D matrix showed the presence of the pan-neuronal marker NeuroChrom and presynaptic synaptophysin 1 (SYP1) puncta (Fig. 1B) as well as abundant expression of β-3-tubulin (Fig. 1C) throughout the 3D co-culture, confirming the generation of neuronal extensions and presynapses. Importantly, GFAP-positive astrocytes with multiple long processes were observed dispersed throughout the 3D neuronal network (Fig. 1B, C), indicating the integration of astrocytes in the 3D co-cultures. These data demonstrate the utility of this protocol for the generation of 3D co-cultures of human neurons and astrocytes.

### Morphologically complex astrocytes interact with neurons in 3D co-culture

**Fig. 2:**
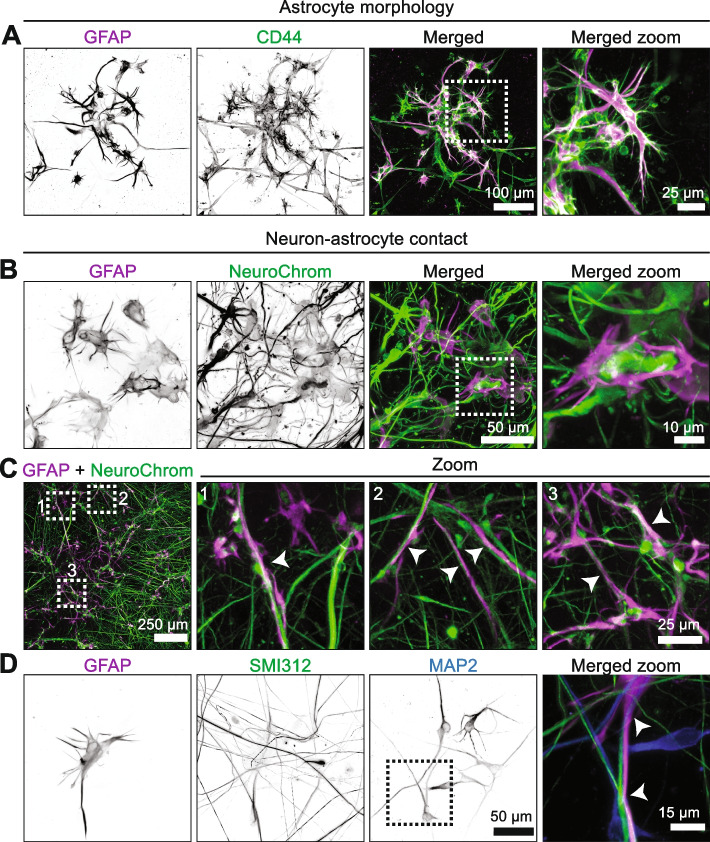
Morphologically complex astrocytes interact with neurons. HiPSC-derived neurons and human primary astrocytes were co-cultured in 3D for 4 weeks, and astrocyte morphology and interaction with neurons was assessed by confocal microscopy. **A** 160 µm-thick maximum intensity confocal image projection of neurons and astrocytes in 3D co-culture. Immunostaining was performed for astrocytes (GFAP, magenta; CD44, green). Single channels are shown in greyscale. A zoom of the boxed area indicated in the merged image is shown. **B** 112 µm-thick maximum intensity confocal image projection of neurons and astrocytes in 3D co-culture. Immunostaining was performed for neurons (NeuroChrom, green) and astrocytes (GFAP, magenta). Single channels are shown in greyscale. A zoom of the boxed area indicated in the merged image is shown. See Supplementary Video 1 for a confocal z-stack. **C** 105 µm-thick maximum intensity confocal image projection of neurons and astrocytes in 3D co-culture. Immunostaining was performed for neurons (NeuroChrom, green) and astrocytes (GFAP, magenta). A merged image is shown, in which three numbered boxed areas are shown as zoomed areas. In the zoomed images, the arrows indicate astrocytes extending upon neuronal processes. **D** 78 µm-thick maximum intensity confocal image projection of axons, dendrites and astrocytes in a 3D co-culture. Immunostaining was performed for axons (SMI312, green), dendrites (MAP2, blue) and astrocytes (GFAP, magenta). Single channels are maximum intensity projections and shown in greyscale. The boxed area indicated in the MAP2 channel is shown as a merged image and the arrows indicate an astrocyte interacting with axons and dendrites

Astrocytes in vivo display a complex morphology and contain numerous processes [17]. GFAP immunoreactivity may not label all astrocytes and their thinner processes [18], therefore we performed additional immunostaining of the glial membrane marker CD44 to investigate astrocyte morphology in more detail. In agreement with a previous study in post-mortem human brain [19] these data confirmed that not all CD44-positive astrocytes express GFAP (Fig. 2A). Also, these data demonstrate that extensive thin CD44-positive astrocytic processes are present in the 3D co-culture. Finally, and in accordance with previous studies [20, 21], these data show that human astrocytes in our 3D co-culture exhibited a bipolar or stellate morphology (Fig. 2A), akin to the morphology of fibrous astrocytes in situ [22] that is typically not found in 2D co-culture [11]. These data demonstrate that human astrocytes in the 3D co-culture obtain the morphological complexity similar to those in the human brain.

In the human brain, astrocytes contact neuronal cell bodies and synapses via their extensive processes [17]. Although immunostaining for CD44 labeled more astrocytes and thinner processes compared to GFAP (Fig. 2A), it also increased non-specific staining of larger spherical structures, likely derived from membranous debris. Therefore, to minimize non-specific staining for the assessment of neuron-astrocyte contact in the 3D co-cultures we performed double immunofluorescent staining of GFAP and NeuroChrom to label astrocytes and neurons, respectively. Confocal microscopy demonstrated that astrocytes are closely associated with neurons and often enwrap neuronal somas and neurites (Fig. 2B and Supplementary Video 1). In addition, the astrocytes were regularly found aligned with neuronal processes (Fig. 2C and Supplementary Video 1). To further assess astrocyte contact with axons and dendrites, we performed immunofluorescence staining and confocal microscopy for axons (SMI312) and dendrites (MAP2). First, this showed the differential localization of axonal and dendritic markers in neuronal extensions (Fig. 2D), demonstrating that hiPSC-derived neurons in the 3D co-culture acquire polarity of axons and dendrites within 4 weeks. In addition, this demonstrated that astrocytic GFAP-positive processes align in close contact with both axon and dendrite tracts. (Fig. 2D). Altogether, these data demonstrate that human astrocytes in the 3D co-culture display an in vivo*-*like morphology and interact with hiPSC-derived neurons.

### Neurons form synapses and display spontaneous calcium transients in 3D co-culture

**Fig. 3:**
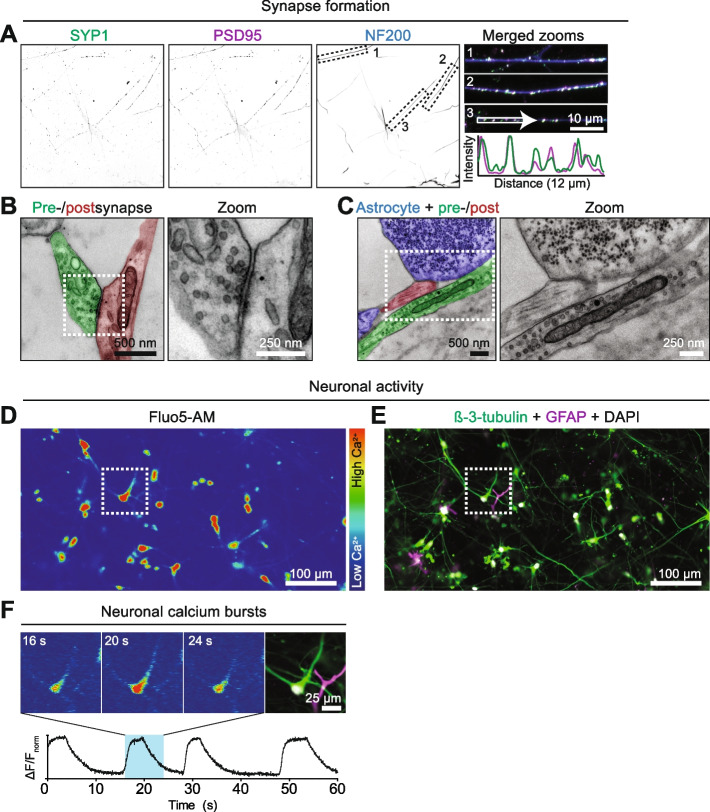
Synapse formation and neuronal activity. Synapse formation (**A-C**) and calcium activity (**D-F**) was assessed in 4-week old 3D human neuron/astrocyte co-cultures **A** 12 µm-thick maximum intensity confocal image projection of axons, pre- and postsynapses in a 3D co-culture. Immunostaining was performed for axons (NF200, blue), pre- (SYP1, green) and postsynapses (PSD95, magenta). Single channels are shown in greyscale and zooms of the numbered boxed areas, indicated in the NF200 panel, are shown as merged. Intensity profiles of SYP1 (green) and PSD95 (magenta) were determined along the line segment indicated in merged zoom 3 to show co-localization of pre- and postsynaptic compartments. **B** Transmission electron micrograph of a pre- and postsynapse (pseudocolour in green and red, respectively). The boxed area indicated in the pseudocoloured image is shown as a greyscale image. **C** Transmission electron micrograph of an astrocyte (blue) in contact with a pre- and postsynaptic compartment (pseudocolour in green and red, respectively). Note that the astrocyte contains electron-dense glycogen granules. The boxed area indicated in the pseudocoloured image is shown as a greyscale image. A 4-week old 3D human neuron/astrocyte co-culture **D, F** was loaded with Fluo5-AM for calcium imaging and **E** subsequently labeled with β-3-tubulin and GFAP to assess the neuronal/astrocytic source of the calcium signal. **D** A 10-min maximum fluorescence intensity projection of Fluo5-AM. The heatmap corresponds to the calcium concentration. See Supplementary Video 2 for calcium signal over time. **E** After calcium imaging, immunofluorescent staining was performed for neurons (β-3-tubulin, green) and astrocytes (GFAP, magenta), and nuclei were visualized using DAPI (greyscale). **F** Calcium bursting in one cell indicated by the boxed area in D at three different time points (t = 16, 20 and 24 s) and the corresponding immunofluorescent image of the boxed area in E. The graph shows the Fluo5-AM fluorescence intensity of this cell (cell 2 in Supplementary Fig. 1A-C) over 1 min, with the lowest and highest value set to 0 and 1 respectively, and the blue box corresponds to the timeframe between t = 16 and 24 s for which the bursting activity is shown. Please note that no calcium signal was observed in the astrocyte. See Supplementary Fig. 1 for the neuronal/astrocytic annotation of calcium traces

Synapses permit the specific activity and function of the neuronal network and the interaction between astrocytes and synapses is pivotal for synapse function [23]. We demonstrated the formation of presynaptic structures throughout the 3D co-culture (Fig. 1B). To further investigate the formation and contact between pre- and postsynapses in the 3D co-culture, double immunofluorescent staining and subsequent confocal microscopy using presynaptic SYP1 and postsynaptic density protein 95 (PSD95) was performed. SYP1 and PSD95 positive puncta colocalized onto axons (NF200; Fig. 3A), demonstrating that neurons in this 3D co-culture form a morphologically mature synapse pattern [24, 25]. To confirm the contact between pre- and postsynaptic compartments in the 3D co-culture at higher resolution we next optimized the processing of 3D co-cultures for transmission electron microscopy (EM) imaging. EM imaging showed presynaptic terminals containing synaptic vesicles connected to an aligned postsynaptic plasma membrane with an electron-dense postsynaptic density (Fig. 3B). This confirms the formation of pre- and postsynapses in the 3D co-culture and demonstrates the compatibility of this system with ultrastructural analysis. Interestingly, EM also demonstrated cellular processes containing intracellular glycogen granules, a characteristic of astrocytes [26], in contact with neuronal compartments (Fig. 3C). This included close contact with presynaptic terminals containing synaptic vesicles connected to an aligned postsynaptic plasma membrane, suggestive of a pre- and postsynaptic terminal in a tripartite synapse. These data demonstrate synapse formation and further confirm the interaction between astrocytes and neurons in the 3D human co-culture.

We next examined whether hiPSC-derived neurons in the 3D co-culture display calcium transients, indicative of neuronal activity [27]. Using live-cell imaging of Fluo-5 AM, a calcium-sensitive dye, spontaneous calcium transients were observed and recorded for 10 min (Fig. 3D and Supplementary Video 2). After live-cell imaging the sample was fixed and processed for immunofluorescence to determine the cellular source of the 15 calcium traces (Supplementary Fig. 1A-C) using β-3-tubulin and GFAP to distinguish neurons and astrocytes, respectively (Fig. 3E). This showed that neurons are the primary source of the recorded calcium traces (12/15 cells, 2 cells unidentified; Supplementary Fig. 1C). One astrocyte displayed a very slow rise in Fluo-5 AM intensity throughout the 10 min-record, suggesting the gradual internalization of the dye rather than astrocytic calcium activity. For a representative active neuron, the burst-to baseline calcium transients lasted 3–5 s (Fig. 3F), in accordance with the timescales previously observed for human neurons cultured in 3D microfluidic platforms [20, 28]. These data demonstrate that the human neurons in the 3D co-culture form synapses and display neuronal activity.

### Intraneuronal tau aggregation in 3D co-culture

**Fig. 4:**
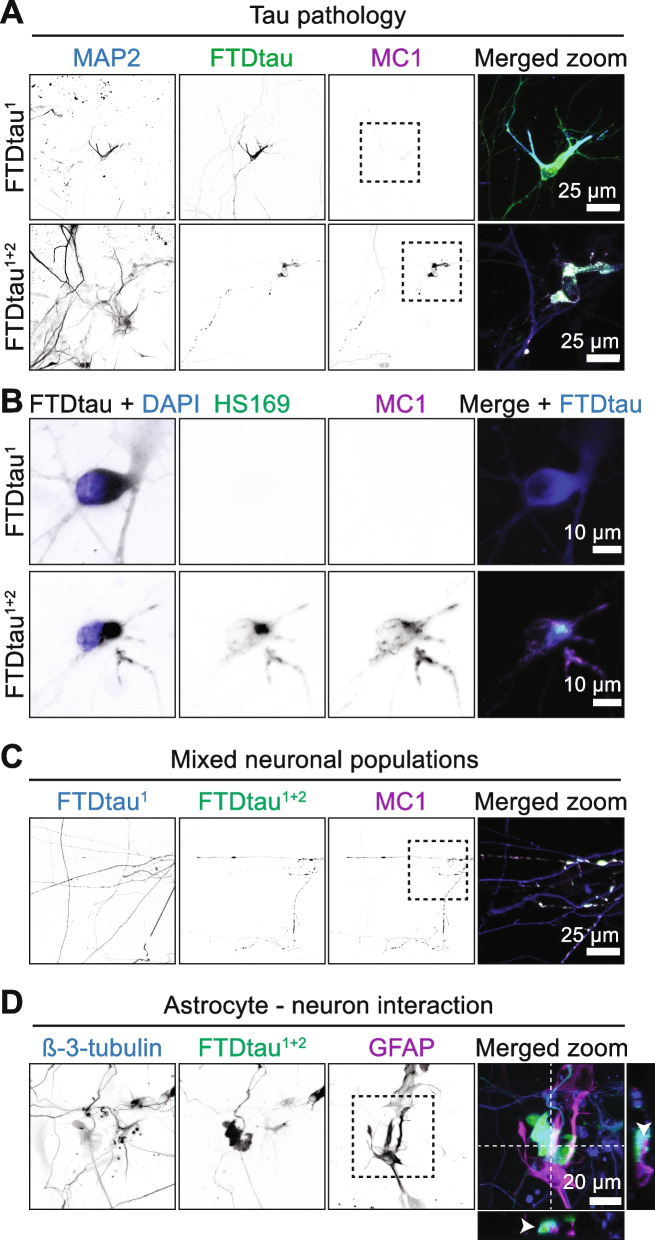
Seed-independent intraneuronal tau aggregation. Neural progenitors were transduced with mCherry-tagged FTDtau^1^ or EGFP-tagged FTDtau^1+2^, mixed separately (**A, B, D**) or combined (**C**) with human primary astrocytes and tau pathology was assessed at week 4. **A** 12 µm-thick maximum intensity confocal image projections of 3D-cocultures expressing EGFP-tagged FTDtau^1^ or FTDtau^1+2^ (green), immunostained for neurons (MAP2, blue) and a pathological conformation of tau (MC1, magenta). Single channels are shown in greyscale. A zoom of the boxed area indicated in the MC1 channel is shown as merged image and shows overlap of FTDtau^1+2^ and MC1 within a MAP2-positive neuron. **B** 11 µm-thick maximum intensity confocal image projection of 3D co-cultures expressing EGFP-tagged FTDtau^1^ or FTDtau^1+2^, immunostained for a pathological conformation of tau (MC1), co-labelled for stacked beta-sheets (HS169). Nuclei were visualized using DAPI (blue) and are merged with FTDtau (grey). The HS169 and MC1 single channels are shown in greyscale and the merged image shows that only FTDtau^1+2^ (blue) overlaps with MC1 (magenta) and HS169 (green). **C** 10 µm-thick maximum intensity confocal image of a 3D neuron/astrocyte co-culture with neurons expressing either mCherry-tagged FTDtau^1^ (blue) or EGFP-tagged FTDtau^1+2^ (green), immunostained for a pathological conformation of tau (MC1, magenta). A zoom of the boxed area indicated in the MC1 channel is shown as merged image and shows that only FTDtau^1+2^ overlaps with MC1. **D** 50 µm-thick maximum intensity confocal image projections of a 3D-coculture expressing EGFP-tagged FTDtau^1+2^ (green), immunostained for neurons (β-3-tubulin, blue) and astrocytes (GFAP, magenta). Single channels are shown in greyscale. A zoom of the boxed area indicated in the GFAP channel is shown as merged image. The arrows in the orthogonal views show an astrocytic extension in contact with a FTDtau^1+2^-containing neuron

In tauopathies, tau aggregates are present in neurons and contribute to neurodegeneration in a cell-autonomous and non-autonomous manner. To investigate whether the 3D co-culture model can be employed for the study of cell type-specific pathology, we introduced tau pathology specifically in neurons. The introduction of tau containing two FTD-associated mutations P301L and S320F (FTDtau^1+2^) previously enabled seed-independent tau aggregation in 3D rodent brain slice cultures [29]. Viral transduction of neural progenitors with FTDtau^1^ (P301L) or FTDtau^1+2^ (P301L + S320F) was performed one day before 3D co-culturing with human astrocytes. Confocal microscopy showed that expression of FTDtau^1+2^ but not FTDtau^1^ resulted in the formation of MC1-positive, pathological tau in MAP2-positive neurons in the 3D co-culture within 4 weeks (Fig. 4A). Moreover, MC1-positive FTDtau^1+2^ was positive for the thiophene dye HS169 [30] (Fig. 4B), demonstrating the formation of β-sheet-rich tau aggregates.

The protocol also allows the introduction of differentially manipulated neuronal populations to investigate neuron-neuron interactions. As a proof-of-concept a mixture of neurons expressing FTDtau^1^- and FTDtau^1+2^ with distinct fluorescent tags (EGFP and mCherry) was introduced in 3D co-culture with human astrocytes. These data show that FTDtau^1^- and FTDtau^1+2^-expressing neurons could be clearly distinguished and importantly only the latter population developed MC1-positive tau pathology (Fig. 4C), confirming that the phenotype of different tau variants (Fig. 4A) is reproduced in a mixed co-culture.

Tau aggregation in the 3D co-culture is independent on exogenous seeds and therefore models the effects of intraneuronal tau pathology. This makes the model potentially suitable for the study of cell non-autonomous disease mechanisms. Interestingly, interactions of GFAP-positive astrocytes with neurites and cell bodies of β-3-tubulin-positive neurons containing FTDtau^1+2^ are present (Fig. 4D). This indicates that the interaction between neurons and astrocytes is intact. Altogether these data provide proof-of-concept for the utility of the model to study early, cell non-autonomous disease mechanisms in the pathogenesis of tauopathies.

## Discussion

Here we established and validated a novel 3D human neuron/astrocyte co-culture model of tau pathology. First, we report a step-by-step procedure to generate 100–200 µm-thick microwell co-cultures of hiPSC-derived neurons and primary human astrocytes. Using live, confocal as well as electron microscopy techniques, we demonstrate that the neurons in the 3D co-culture form pre- and postsynapses, display spontaneous calcium transients, and are in close contact with astrocytes. Second, by the introduction of two pathogenic tau mutations we are the first to demonstrate seed-independent tau aggregation in a human 3D co-culture.

We present a step-by-step protocol to generate miniaturized 3D co-cultures. These 3D co-cultures are more robust than traditional 3D organoids that may suffer from heterogeneity in size and cellular composition, limited throughput, and culturing and processing procedures that may be time-consuming or costly [31]. The 3D co-cultures presented here consist of homogeneous populations of hiPSC-derived neurons and human primary astrocytes and are compatible with standard imaging techniques. First, hiPSC-derived neural progenitors are mixed in a defined ratio with primary human astrocytes and subsequently embedded within an ECM, resulting in a homogeneous distribution and consistent density of cells. Second, since the ECM polymerizes at the bottom of 96-well microplates, it shapes as a thin cylinder that permits easy media refreshment and is structurally supported by the well sides. The 3D ECM remained stable for at least 4 weeks but preliminary data indicate that co-cultures are stable at 8 weeks, suggesting that they can also be used for studies that require prolonged culturing. Third, by utilizing 96-well plates we miniaturized the system and the high-clarity foil bottom ensured its compatibility with standard imaging techniques with high signal to noise. This format also permits the processing as well as (live) imaging of the 3D co-cultures directly in the plate, as opposed to organoids that typically require collection, embedding and sectioning. Specific 3D culture plates are commercially available, in which cells are either pipetted into microfluidic channels [20] or plated on top of a precast ECM matrix [32]. In contrast, in the current study standard cell culture plates are used, thus reducing technical and financial limitations to broadly implement the protocol.

Both the neurons and astrocytes present mature phenotypes within 4 weeks. Using immunofluorescence, live- and EM imaging techniques, we demonstrate that hiPSC-derived neurons in the 3D co-culture express typical neuronal markers, acquire polarity of axons and dendrites, form pre- and postsynapses and display calcium activity within 4 weeks. In addition, we show that astrocytes in the 3D co-culture are integrated in the neuronal network and display bipolar and stellate morphologies with extensive processes that are not typically found in 2D monoculture. This is consistent with previous studies that showed that rodent [33] as well as human [34] astrocytes adopt a more in vivo*-*like morphology when maintained in a 3D compared to 2D environment [10]. Also, rodent studies have previously demonstrated that morphological complexity of astrocytes is increased by co-culture with neurons [35, 36], largely mediated via connexins [37] and neuroligins [35] in a contact-dependent manner. Similarly, and in agreement with previous observations in an hiPSC-derived organoid system [11], we demonstrate that astrocytes interact with neurons in the 3D co-culture and extend their processes along axon and dendrite tracts. Importantly, astrocytes were found in close contact with neuronal cell bodies as well as with synapses, suggesting the formation of tripartite synapses. Indeed, such contacts are pivotal for astrocytes to control neuronal homeostasis in the brain, which includes the regulation of ion levels, storage and supply of energy, turnover of neurotransmitters as well the formation, function and elimination of synapses [23, 26, 38]. Altogether, the 3D co-culture offers a novel tool to study both cell types and the mechanisms by which they interact in a physiologically relevant 3D environment.

To our knowledge, we are the first to demonstrate intraneuronal tau pathology in a 3D human co-culture model in a seed-independent manner. The study of aging-related diseases such as sporadic tauopathies in hiPSC-derived neurons is challenging as they rarely match the transcriptional and functional maturity of human neurons in vivo [15, 39], and lack postnatal 4R tau isoforms even after 100 days in vitro [40, 41]. As is commonly observed in developing neurons, hiPSC-derived neurons often present with increased levels of tau phosphorylation, but do not develop insoluble aggregates of endogenous tau [40, 42–45]. Altogether, these factors necessitate the overexpression of FTD-associated tau variants to model tau aggregation in hiPSC-derived neurons [46, 47]. The slow spontaneous aggregation rate of these variants, however, in combination with extracellular tau as a possible means of disease propagation [48, 49], led to the development of tau seeding models. In this paradigm, tau aggregation can be induced by exposure to pre-formed tau aggregates (seeds) in rodent [50] and human [46] cell models as well as in transgenic mice [51]. Tau seeding was also implemented for hiPSC-derived neurons embedded in a 7.5-fold lower concentration ECM than used in our model [47], which in our hands was not sufficient to generate a 3D structure. More importantly, seeds can induce cell non-autonomous responses independently of intraneuronal tau pathology [52, 53]. Since this may be a confounding factor for the investigation of how tau pathology *within* neurons affects their interaction with astrocytes in the 3D culture, we adopted a novel approach for seed-independent tau pathology using double mutant FTDtau^1+2^. This approach previously enabled seed-independent tau pathology in rodent brain slice cultures [29] and a 2D human neuron/astrocyte co-culture [8]. In agreement, neuron-specific expression of FTDtau^1+2^ induces intraneuronal MC1-positive tau pathology within 4 weeks in the 3D co-culture. In addition, the pathological inclusions label positive for the thiophene dye HS169 [30], demonstrating the accumulation of stacked beta-sheets that are typical for inclusions in the human brain. Altogether, these data demonstrate the utility of the 3D co-culture to model intraneuronal tau pathology that does not require seeds.

Our system is highly versatile and can easily be adapted to address more specific research questions. For example, the protocol contains a 24-h time window for manipulation with lentiviral transduction before co-culture. This allows the introduction of cell-specific manipulations like overexpression or knock-down of (e.g. disease-associated) proteins without the need for cell type-specific promoters. Moreover, the model can be used for the study of neuron-neuron interactions. As a proof-of-concept, we mixed two neuron populations overexpressing distinctly labelled FTDtau variants, demonstrating the specificity of FTDtau^1+2^ for the formation of tau pathology in the same 3D culture. In tau transgenic mice it was demonstrated that tau pathology can propagate to neurons as well as astrocytes that did not express the transgene [54]. Since in the mixed 3D co-culture of FTDtau^1^- and FTDtau^1+2^-expressing neurons we only observe tau pathology in the latter population, our data suggests that tau pathology in the population with FTDtau^1+2^ did not propagate within the timeline of our experiments. Importantly, despite the close neuron-astrocyte contact, we do not observe transfer of tau (pathology) between neurons and astrocytes in the 3D culture. Moreover, we do not observe overt effects of tau pathology on neurons or astrocytes in the 3D co-culture. This makes the model suitable to study early events in tau pathogenesis that precede neurodegeneration.

A limitation of this study is the qualitative assessment of cellular phenotypes. More subtle and/or cell non-autonomous effects of tau pathology should be addressed in a quantitative manner in future studies. To this end, future efforts should prioritize the development of advanced cell segmentation tools for high-content imaging in 3D. Readouts can include the loss of synapses and neurites, the extent of tau pathology, the transfer of tau between neurons and astrocytes, as well as astrocytic morphology and (inflammatory) marker expression. In addition, single-cell omics techniques can be used to assess the cell-specific neuronal and astrocytic transcriptional responses to intraneuronal tau pathology. The feasibility of this approach to assess cell type-specific disease pathways in 3D cultures was recently demonstrated in a single-cell RNA sequencing study of iPSC-derived organoids, showing that tau mutations lead to cholesterol dysregulation specifically in astrocytes [55], but tau pathology was not studied. Since preliminary data indicate that our 3D co-cultures are also stable at 8 weeks, both these quantitative approaches may be complemented with prolonged culturing.

Our model does not include all cellular components: also other brain cells and in particular microglia play a crucial role in the pathogenesis and progression of tau pathology [56, 57]. Indeed, microglial activation coincides with tau pathology in patients [58–60] and rodent studies have demonstrated a variety of microglial processes that contribute to the progression of the disease, including the phagocytosis of neurons, synapses and tau, as well as the secretion of pro-inflammatory cytokines [61–66]. Rodent models have strongly contributed to our understanding of the (micro)glial component of tauopathies. Although gene expression patterns are remarkably similar between rodent and human microglia, some striking species-specific differences exist in microglial immune regulation [67] and aging [68]. In addition, the cellular and anatomical complexity of in vivo models may limit further mechanistic insight and cell type-specific intervention in the communication between neurons, astrocytes and microglia. On the other hand, 2D in vitro monocultures lack the in vivo complexity to accurately model the physiological and pathological responses of neural cells in general, and microglia in particular [67, 69] similar to astrocytes, as mentioned above. For ß-amyloid formation, a 3D neuron/astrocyte/microglia tri-culture has been developed using immortalized microglia [28], but no 3D tri-culture of tau aggregation exists yet. The 3D co-culture model of tau pathology we present here can be further developed to a 3D tri-culture by incorporating microglia. This approach would create the possibility to investigate interactions between microglia, astrocytes and neurons with tau pathology, and their role in neuroinflammation and neurodegeneration.

## Conclusions

We have developed a 3D human co-culture model with synapse formation, neuronal activity and abundant neuron-astrocyte interactions. Using this model, we are the first to introduce intraneuronal tau pathology in a 3D co-culture in a cell-specific and seed-independent manner. This model therefore provides a suitable miniaturized platform to study cell (non-) autonomous disease mechanisms of tau pathogenesis in a physiologically relevant 3D environment.

## Methods

### Stem cell and primary human astrocyte cultures

A previously generated hiPSC line stably expressing a doxycyclin-inducible *rtTA/Ngn2* [14] was maintained feeder-free on Vitronectin (Stem Cell Technologies) coated plates in TeSR-E8 medium (Stem Cell Technologies; 1X TeSR-E8 supplement (Stem Cell Technologies), 50 U/mL Pen/strep (Gibco)) supplemented with G418 (50 µg/mL; Sigma) and puromycin (0.5 µg/mL; Sigma) at 37 °C/5% CO_2_ under hypoxic conditions. Colonies were fed daily and double-volume feeding allowed for weekend-free culturing. Once or twice a week, colonies were passaged by non-enzymatic dissociation with Gentle Cell Dissociation Reagent (Stem Cell Technologies) and replated in the presence of Rho-associated, coiled-coil containing protein kinase (ROCK) inhibitor (RI; 10 µM; Selleckchem) for 24 h to promote cell survival. To prevent genomic instability and senescence by prolonged passaging of hiPSCs, cryopreserved cells were thawed 2 weeks prior to use once cells reached passage number 25.

Primary human fetal astrocytes (ScienCell #1800) were cultured in Astrocyte Medium (ScienCell; 1X Growth Supplement, 1X FBS, 100 U/mL Pen/strep (all ScienCell)) on Geltrex (1:100 in DMEM/F12 (both Gibco) coated plates and half of the medium was refreshed twice a week. Astrocytes were passaged once a week by enzymatic dissociation with Accutase (Sigma). Cultures were kept at 37 °C/5% CO_2_ at physiological oxygen conditions and used until passage number 7. To eliminate variability in astrocyte physiology due to genetic background of the donor, all experiments in this study were conducted with cryopreserved cells that were expanded from one vial.

### Human co-culture and neuronal differentiation

The materials and standard operating procedures to generate the 3D human co-cultures are described in Supplementary Document 1. Briefly, two days prior to 3D co-culture (i.e. day -2), hiPSCs were non-enzymatically dissociated with Gentle Cell Dissociation Reagent and plated as single cells (80.000 cells/well) in 6-well culture plates (VWR) precoated with Geltrex (1:100 in DMEM/F12) in TesR-E8 medium with NT3 (10 ng/mL; Peprotech), BDNF (10 ng/mL; Peprotech), RI (10 µM) and doxycycline (2 µg/mL; Sigma) for induction of the Ngn2 transgene. After one day (i.e. day -1), medium was changed to DMEM/F12 (Gibco; 1X N2 supplement, 1X NEAA, 100 U/mL Pen/strep (all Gibco)) with NT3 (10 ng/mL), BDNF (10 ng/mL) and doxycycline (2 µg/mL). At the day of co-culture (i.e. day 0), Ngn2 neural progenitors and primary human astrocytes were treated with Accutase for detachment. Prior to detachment, Accutase was aspirated and cells were dissociated separately in Neurobasal Medium (Gibco; 1X B27 + VitA, 1X Glutamax, 100 U/mL Pen/strep (all Gibco)) with NT3 (10 ng/mL), BDNF (10 ng/mL), RI (10 µM) and doxycyclin (2 µg/mL). After centrifugation the supernatant was removed and the co-culture cell suspension (30.000 neural precursors with 5.000 human astrocytes in 25 µL per well) was mixed with undiluted Geltrex (50% v/v final concentration) and kept on ice to prevent polymerization. Co-culture suspensions were plated in black 96-well culture plates (Greiner #655,090) and, once a week, half (week 1 and 2) or two-third (week 3) of the medium was refreshed until week 4. Only the inner 32 wells of the 96-well plate were used for culturing and empty wells were filled with sterile PBS to minimize evaporation of the culture medium. Throughout the neuronal differentiation process, co-cultures were kept at 37 °C/5% CO_2_ at physiological oxygen conditions. See Supplementary Document 1 for a step-by-step culture procedure.

### Lentiviral transduction

Lentiviral transduction of Ngn2-neural progenitors was performed one day prior to co-culture (i.e. day -1) with medium change to DMEM/F12 containing 4 µL/mL viral solution. At the day of co-culture (i.e. day 0), 24 h after transduction, cells were washed once for 30 min with pre-warmed Neurobasal Medium containing all factors to remove lentiviral particles and thus prevent transduction of astrocytes during subsequent plating. In a 2^nd^ generation lentiviral backbone, the expression of 2N4R FTDtau with an in-frame EGFP or mCherry C-terminal fluorescent tag was driven by a cytomegalovirus promoter. The following FTDtau mutants were used: P301L (FTDtau^1^) and P301L + S320F (FTDtau^1+2^). Lentiviral particles were generated as previously described [70]**.**

### Immunofluorescence staining

Culture media was removed and cells were washed with PBS and fixed with paraformaldehyde (PFA), consisting of 30 min incubation at room temperature (RT) in 4% PFA (Electron Microscopy Sciences) in PBS (pH 7.4). Fixed cultures were washed once with PBS for 30 min and immediately processed for immunostaining or stored at 4 °C. For immunostaining, cells were blocked and permeabilized for 3 h in blocking solution (5% normal goat serum (Thermo Fisher) + 0.1% bovine serum albumin and 0.3% Triton X-100 (both Sigma) in PBS (pH 7.4)), and subsequently incubated with primary antibodies diluted in blocking solution for 1 h at RT followed by overnight at 4 °C. After washing three times in PBS, cells were incubated overnight at 4 °C with Alexa Fluor-conjugated secondary antibodies (488, 546 or 568 and 647) (Invitrogen) diluted 1:1000 in blocking solution. HS169 (1:500, a kind gift of Peter Nilsson) was used during secondary antibody incubation to label stacked beta-sheets [30]. Cells were washed three times in PBS for 15 min at RT, and cell nuclei were stained with 4′,6-diamidino-2-phenylindole (DAPI) (2.5 µg/mL, Sigma) in PBS of the second wash. Confocal images were acquired with a Nikon Eclipse Ti confocal microscope equipped with a 10 × air and 40 × oil immersion objective and controlled by NisElements 4.30 software (Nikon). Z-stacks with a step size between 1 µm (for stacks < 15 µm) and 5 µm (for stacks > 15 µm) were obtained and each parameter, including laser power and detector gain, was kept constant within each experiment for valid comparison*.* The following primary antibodies were used: CD44 (1:100, DSHB, H4C4), Cy3-conjugated NeuroChrom (1:500, Merck, ABN2300C3), GFAP (1:1000, DAKO, Z0334), MAP2 (1:2500, Millipore, AB5543), MC1 (1:500, kind gift of Peter Davies), NF200 (1:200, Sigma, N4142), SMI312 (1:1000, Eurogentec, 312P-050), PSD95 (1:200, Abcam, AB2723), Synaptophysin 1 (1:500, Synaptic Systems, 101,004) and Tuj1 (1:1000, R&D systems, MAB1195). Maximum intensity projected Z-stacks are shown. ImageJ software (National Institute of Health) was used to adapt images for publication. Immunofluorescence data are supported by at least 3 independent experiments.

### Calcium imaging

To define spontaneous calcium transients, cells were incubated with 1 μM Fluo‐5F‐AM (Molecular Probes, F14222) in complete culture medium for 30 min at 37 °C/5% CO_2_. High-speed confocal imaging was performed in resonant mode using the Nikon Eclipse Ti confocal microscope, equipped with a 10 × air objective and controlled by NisElements 4.30 software (Nikon). Images (512 × 256 pixels at 2 × scanner zoom) were acquired for 10 min at 30 Hz on a 37 °C heated stage. Raw intensity values were extracted in ImageJ (National Institute of Health) for each cell source, and the lowest and highest fluorescent signal was scaled between 0 and 1 respectively. Calcium data are supported by 3 independent experiments.

### Electron microscopy

Culture media was removed and cells were washed once with PBS, followed by fixation with 2.5% glutaraldehyde (Merck) in 0.1 M cacodylate buffer, pH7.4, overnight at 4 °C. Samples were washed three times with 0.1 M cacodylate buffer and post-fixed by 1% OsO_4_/1% Ru(CN)_6_ for 90 min at RT. After washing with 0.1 M cacodylate buffer, a series of increasing ethanol concentrations (30%-100%) were used to dehydrate the samples. Samples were embedded in EPON and polymerized for 48 h at 65 °C. The EPON-embedded samples were removed from the substrate by breaking the 96-well plate in a vise. Regions with highest cell density were determined by light microscopy and ultrathin sections of (80 nm) were cut using an Ultracut UCT microtome (Leica). Sections were collected on single-slot, formvar-coated copper grids and counterstained with 0.5% uranyl acetate for 25 min and lead cytrate for 3 min at RT in an automated section stainer (Leica). Images were captured on a JEOL 1010 transmission EM at 60 kV with a side-mounted CCD camera (Modera, EMSIS) controlled by the iTEM software (EMSIS GmbH, Germany) at 40 K or 80 K magnification. EM data are supported by 2 independent experiments.

## Supplementary Information

**Additional file 1: Supplementary Figure 1.** Annotation of calcium activity by immunofluorescence. A 4-week old 3D human neuron/astrocyte co-culture was loaded with Fluo5-AM for calcium imaging and subsequently labeled with β-3-tubulin and GFAP to assess the neuronal/astrocytic source of the calcium signal. (A) A 10-minute maximum fluorescence intensity projection of Fluo5-AM. The heatmap corresponds to the calcium concentration. The numbers correspond to the cells in B and calcium traces in C. See Supplementary Video 2 for calcium signals over time. (B) After calcium imaging, immunofluorescent staining was performed for neurons (β-3-tubulin, green) and astrocytes (GFAP, magenta), and nuclei were visualized using DAPI (greyscale). The numbers correspond to the individual calcium signals in A and traces in C. (C) The graph shows the Fluo5-AM fluorescence intensity of the cells numbered in B cell over 10 minutes, with the lowest and highest value set to 0 and 1 respectively. Intracellular calcium transients were annotated using immunofluorescence for neurons (green) or astrocytes (purple). For two traces (cell 10 and 12), cellular sources were unidentified (black) due to the large overlap of β-3-tubulin and GFAP. Note that the calcium signal of one neuron (cell 9) remains high, possibly by cell death during imaging, as well as that the signal in one astrocyte (cell 15) slowly increases, possibly by passive uptake of the Fluo5-AM dye. 

**Additional file 2: Supplementary Document 1.** Detailed list of reagents and standard operating procedures to generate the 3D human neuron/astrocyte co-cultures.

**Additional file 3: Supplementary Video 1.** Astrocytes enwrap neuronal cell bodies and neurites. Z-stack confocal video of neurons and astrocytes in a 112 µm-thick 3D co-culture displayed in Figure 2B. Immunostaining was performed for neurons (NeuroChrom, green) and astrocytes (GFAP, magenta).

**Additional file 4: Supplementary Video 2. **Calcium activity in 3D neuron/astrocyte co-culture. A 4-week old 3D human neuron/astrocyte co-culture was loaded with Fluo5-AM for calcium imaging. Confocal video showing Fluo5-AM fluorescence over time for 10 minutes in the 3D co-culture shown in Figure 3D and Supplementary Figure 1A. 

## Data Availability

The data that support the findings of this study are available from the corresponding author upon reasonable request.

## Abbreviations

2D: Two-dimensional
3D: Three-dimensional
AD: Alzheimer’s disease
ECM: Extracellular matrix
EM: Electron microscopy
FTD: Frontotemporal dementia
hiPSCs: Human induced pluripotent stem cells
Ngn2: Neurogenin 2
PSD95: Post-synaptic density protein 95
SYP1: Synaptophysin 1
