# Supplementary material for: A 3D human co-culture to model neuron-astrocyte interactions in tauopathies
Source: Biol Proced Online. 2023 Feb 23;25:4. doi: 10.1186/s12575-023-00194-2 (PMC9948470; doi:10.1186/s12575-023-00194-2)
Supplement: Supplementary file 1 — Additional file 1: Supplementary Figure 1. Annotation of calcium activity by immunofluorescence. A 4-week old 3D human neuron/astrocyte co-culture was loaded with Fluo5-AM for calcium imaging and subsequently labeled with β-3-tubulin and GFAP to assess the neuronal/astrocytic source of the calcium signal. (A) A 10-minute maximum fluorescence intensity projection of Fluo5-AM. The heatmap corresponds to the calcium concentration. The numbers correspond to the cells in B and calcium traces in C. See Supplementary Video 2 for calcium signals over time. (B) After calcium imaging, immunofluorescent staining was performed for neurons (β-3-tubulin, green) and astrocytes (GFAP, magenta), and nuclei were visualized using DAPI (greyscale). The numbers correspond to the individual calcium signals in A and traces in C. (C) The graph shows the Fluo5-AM fluorescence intensity of the cells numbered in B cell over 10 minutes, with the lowest and highest value set to 0 and 1 respectively. Intracellular calcium transients were annotated using immunofluorescence for neurons (green) or astrocytes (purple). For two traces (cell 10 and 12), cellular sources were unidentified (black) due to the large overlap of β-3-tubulin and GFAP. Note that the calcium signal of one neuron (cell 9) remains high, possibly by cell death during imaging, as well as that the signal in one astrocyte (cell 15) slowly increases, possibly by passive uptake of the Fluo5-AM dye. [file 12575_2023_194_MOESM1_ESM.pdf]

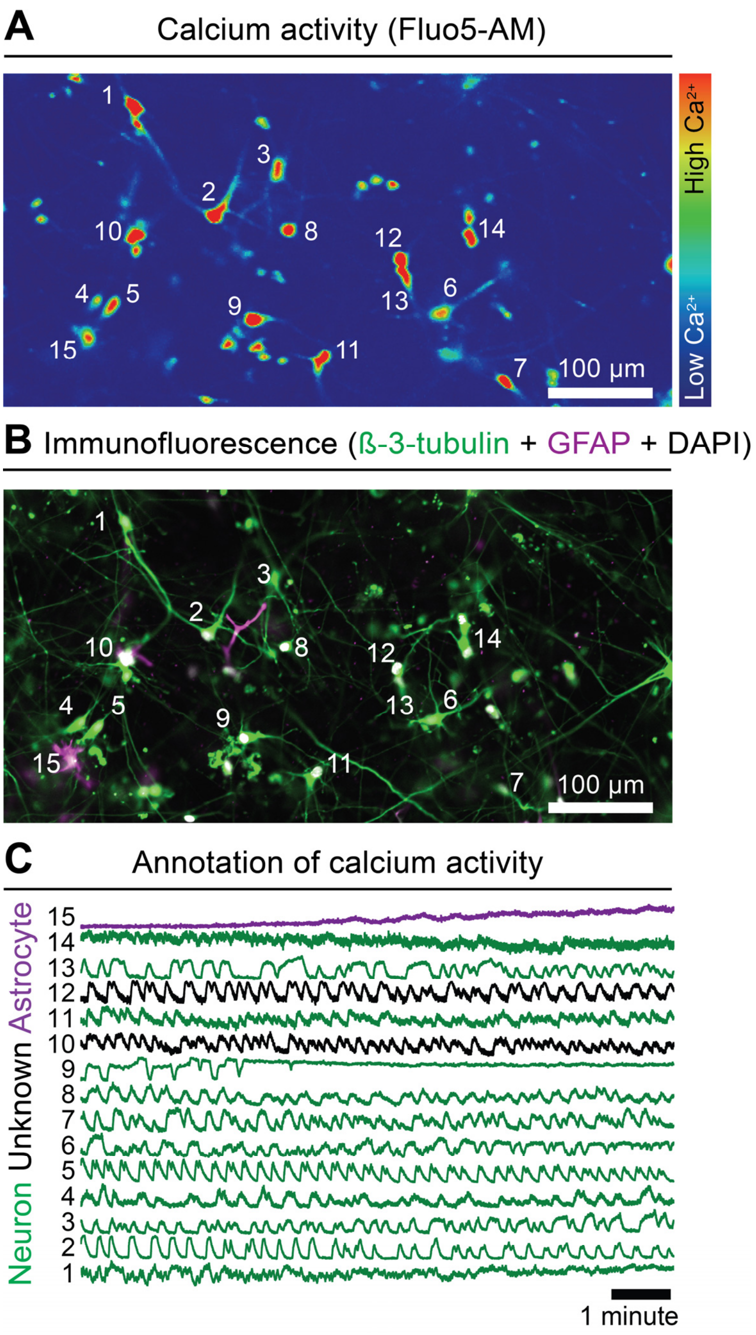

Supplementary Figure 1
Annotation of calcium activity by immunofluorescence
A 4-week old 3D human neuron/astrocyte co-culture was loaded with Fluo5-AM for calcium imaging and subsequently labeled with β-3-tubulin and GFAP to assess the neuronal/astrocytic source of the calcium signal. **(A)** A 10-minute maximum fluorescence intensity projection of Fluo5-AM. The heatmap corresponds to the calcium concentration. The numbers correspond to the cells in B and calcium traces in C. See Supplementary Video 2 for calcium signals over time. **(B)** After calcium imaging, immunofluorescent staining was performed for neurons (β-3-tubulin, green) and astrocytes (GFAP, magenta), and nuclei were visualized using DAPI (greyscale). The numbers correspond to the individual calcium signals in A and traces in C. **(C)** The graph shows the Fluo5-AM fluorescence intensity of the cells numbered in B cell over 10 minutes, with the lowest and highest value set to 0 and 1 respectively. Intracellular calcium transients were annotated using immunofluorescence for neurons (green) or astrocytes (purple). For two traces (cell 10 and 12), cellular sources were unidentified (black) due to the large overlap of β-3-tubulin and GFAP. Note that the calcium signal of one neuron (cell 9) remains high, possibly by cell death during imaging, as well as that the signal in one astrocyte (cell 15) slowly increases, possibly by passive uptake of the Fluo5-AM dye.
