# Supplementary material for: A 3D human co-culture to model neuron-astrocyte interactions in tauopathies
Source: Biol Proced Online. 2023 Feb 23;25:4. doi: 10.1186/s12575-023-00194-2 (PMC9948470; doi:10.1186/s12575-023-00194-2)
Supplement: Supplementary file 2 — Additional file 2: Supplementary Document 1. Detailed list of reagents and standard operating procedures to generate the 3D human neuron/astrocyte co-cultures. [file 12575_2023_194_MOESM2_ESM.pdf]

Supplementary Document 1

Reagents

Materials and Reagents

1. 1.5 mL tube (Eppendorf, catalog number: 0030108051)
2. 10 mL pipette (Sarsted, catalog number: 861254001)
3. 10 μL filter tips (Thermo Fisher, catalog number: 11977714)
4. 100 μL filter tips (Thermo Fisher, catalog number: 11953466)
5. 1000 μL filter tips (Thermo Fisher, catalog number: 11973466)
6. 5 mL pipette (Sarsted, catalog number: 861253001)
7. 6 well plate (VWR, catalog number: 734-2323)
8. Accutase (Sigma, catalog number: SF006)
9. Aluminium foil
10. B27 with Vitamin A (Life Technologies, catalog number: 17504001)
11. Black 96-wells plate (Greiner, catalog number: 655090)
12. Brain-derived neurotrophic factor (BDNF) (Prospec, catalog number: Cyt 207)
13. C-Chip Burker Chamber (NanoEntek, catalog number: DHC-B01)
14. DMEM/F12 without L-glutamine (Life Technologies, catalog number: 21331020)
15. Doxycyclin (Sigma, catalog number: D98195-5g)
16. Geltrex (Life Technologies, catalog number: A1413302)
17. Gentle Cell Dissociation Reagent (StemCell Technologies, catalog number: 07174)
18. Glutamax (Life Technologies, catalog number: 35050038)
19. MEM-NEAA (Life Technologies, catalog number: 11140035)
20. Multichannel Pipette
21. Multichannel reservoir (VWR, catalog number: 613-1174)
22. N2 supplement (Life Technologies, catalog number: 17502048)

23. Neurobasal medium (Life Technologies, catalog number: 21103049)
24. Neurotrophin 3 (NT3) (Peprotech, catalog number: 450-03-100ug)
25. Penicillin-Streptomycin (P/S) (Life Technologies, catalog number: 15140122)
26. Phosphate buffered saline (PBS), sterile
27. ROCK-inhibitor (RI) (Selleckchem, catalog number: S1049)
28. TesR-E8 medium with supplement (StemCell Technologies, catalog number: 05990)
29. Trypan Blue (Life Technologies, catalog number: 15250061)

Base media

1. hiPSC medium
   47.75 mL TesR-E8 medium
   2 mL TesR-E8 supplement
   0.25 mL P/S

2. DMEM
   48.5 mL DMEM/F12 without L-glutamine
   500 µL N2 supplement
   500 µL MEM-NEAA
   500 µL P/S

3. Neurobasal medium
   48 mL Neurobasal medium
   1 mL B27 with Vitamin A
   500 µL Glutamax
   500 µL P/S

Procedures

Prior to experiment: coat dishes with Geltrex

1. Per well in a 6-well plate, dilute 10 µL Geltrex in 1 mL DMEM/F12 (1:100 final dilution).
2. Coat the desired number of wells in a 6-well plate with this solution (1 mL/well). Gently tap the plate to ensure a homogenous spreading of the coating.
3. Incubate the plate in a humidified 37°C incubator for at least 4 hours.
4. If necessary, seal the coated plate with parafilm and aluminium foil for storage at 4°C. Coated plates stored at 4°C can be used up to 2 weeks.

Day -2: plate hiPSCs as single cells for induction to neural precursors

1. Let the Geltrex-coated 6-well plate come to room temperature.
2. Prepare:
   a. *Day -2 medium*: hiPSC medium + 10 ng/mL NT3 + 10 ng/mL BDNF + 2 µg/mL Doxycyclin + 10 µM RI, prewarmed in waterbath:
      i. 5 mL for suspension and centrifugation
      ii. 1 mL for resuspension
      iii. 2 mL per well of a 6-well plate for cell plating
   b. 1 sterile 1.5 mL Eppendorf tube with 10 µL Trypan Blue
3. Aspirate the spent medium of hiPSCs with a suction needle or 10 mL stripette.
4. Gently add 1000 µL Cell Dissociation Buffer with a P1000 pipette and transfer the plate to a humidified, low oxygen incubator at 37°C and 5% CO_2. Incubate for 7-9 minutes, until gaps appear between cells at the border and center of the colonies.
5. Gently aspirate the Cell Dissociation Buffer with a P1000 pipette
6. Detach the colonies by flushing with 1 mL *Day -2* medium with a P1000 pipette. Make single cells by gentle up and down pipetting against the corner of the well, but avoid bubble formation. Transfer the cell suspension into the leftover 4 mL *Day -2 medium*. Rinse the dish with 1 mL *Day -2 medium* to collect all cells.
7. Centrifuge the 5 mL cell suspension for 7 minutes at 340 x g at room temperature.

8. Aspirate the supernatant with a suction needle or 10 mL stripette. Resuspend the cell pellet into 1 mL *Day -2 medium*, make single cells by up and down pipetting against the bottom of the tube, but avoid bubble formation.
9. Count live cells using Trypan Blue and C-Chip Burker chamber following manufacturer's instruction.
10. Plate 80.000 live cells in 2mL *Day -2 medium* per well. Incubate the plate overnight in a humidified, regular oxygen incubator at 37°C and 5% CO_2.

Day -1: change medium

1. Prepare 2 mL *Day -1 medium* per well of a 6-well plate: DMEM medium + 10 ng/mL NT3 + 10 ng/mL BDNF + 2 µg/mL Doxycyclin.
2. Aspirate the spent medium of NGN2 neural progenitors with a suction needle or 10 mL stripette.
3. Gently add 2 mL Day -1 medium per well with a stripette. Incubate the plate overnight in a humidified, regular oxygen incubator at 37°C and 5% CO_2.
4. For the next day, thaw Geltrex overnight at 4°C. Undiluted Geltrex can be stored at -80°C. After thawing, aliquot can be stored at 4°C for 2 weeks.

Day 0: suspend neural precursors and human astrocytes in a 3D matrix

1. Let Accutase come to room temperature.
2. Store Geltrex on ice.
3. Prewarm a 96-well plate in a humidified, regular oxygen incubator at 37°C and 5% CO_2.
   - Note: for certain (confocal) microscopes, plates with a recessed bottom (e.g. Greiner catalog number 655866) may be needed.
4. Prepare:
   a. Sterile PBS, 200 uL per empty well to prevent evaporation in the 96-well plate, prewarmed in waterbath
   b. Co-culture medium: Neurobasal medium + 10 ng/mL NT3 + 10 ng/mL BDNF + 2 µg/mL Doxycyclin + 10 µM RI, prewarmed in waterbath

    i. 2x 5 mL for suspension and centrifugation
    ii. 175 µL per well for 3D co-culture in a 96-well plate
  c. 2 sterile 1.5 mL Eppendorf tubes with 19 µL Trypan Blue

5. Aspirate the spent medium of NGN2 neural precursors and human astrocytes.
6. Gently add 1 mL Accutase per well to neuronal precursors and human astrocytes with a stripette. Transfer the plates to a humidified, regular oxygen incubator at 37°C and 5% CO_2. Incubate precursors for 3-5 minutes and human astrocytes for 5-7 minutes.
   - Note: shorter incubation is needed for neural precursors since they detach more quickly compared to human astrocytes at confluency.
7. Gently aspirate Accutase with a P1000 pipette, prevent aspiration of cells.
8. Detach the precursors and human astrocytes by flushing with 1 mL *Co-culture medium* with a P1000 pipette. Make single cells by gentle up and down pipetting against the corner of the well, but avoid bubble formation. Transfer the cell suspensions into their respective 4 mL *Co-culture medium*. Rinse both dishes again with 1 mL of their respective cell suspension in *Co-culture medium* to collect all cells.
9. Centrifuge both cell suspensions for 7 minutes at 340 x g at room temperature.
10. Aspirate most of the supernatant with a suction needle or 10 mL stripette. Resuspend the cell pellets with the residual supernatant (roughly 30-80 µL), gently make single cells by up and down pipetting against the bottom of the tube, but avoid bubble formation.
   - Note: resuspension in the residual supernatant is needed to achieve high density of cells that is needed for 3D culture.
11. Dilute 1 µL of the cell suspensions separately in 19 µL Trypan Blue (1:20 dilution). Count live cells using a C-Chip Burker chamber following manufacturer's instruction
   - Note: for counting cells, determine the volume of the cell suspension by aspirating excess volume, adjust to push out the air and read out the volume at the volumeter.
12. Mix 30.000 neural precursors with 5.000 human astrocytes per well. Top up with *Co-culture medium* to 25 µL total volume per well. Put this cell suspension and undiluted Geltrex on ice.

- Note: prepare volume for 1-2 extra wells to account for pipetting error.

13. Dilute cell suspension 1:2 with undiluted Geltrex (50% v/v final concentration). Gently mix cell suspension, but avoid bubble formation. Put mix on ice.
14. Pipet 50 µL of this cell/Geltrex mix into the corner of a well in a prewarmed 96-well plate with a P200 pipette, avoid bubble formation. Gently tap the plate to ensure the suspension homogeneously spreads in the well.
    - Note: Since 50% Geltrex polymerizes quickly once warmed to 37°C, pipetting by multichannel may be needed to plate large numbers of wells or plates.
    - Note: Reverse pipetting greatly prevents bubble formation but may require the preparation of a larger total volume, see note in step 12.
15. Incubate the plate for 1 hour in a humidified, regular oxygen incubator at 37°C and 5% CO_2.
16. Gently add 150 µL prewarmed *Co-culture medium* to each 3D suspension (200 µL total volume per well) with a P1000 pipette. Add 200 µL prewarmed, sterile PBS to each empty surrounding well with a P1000 or multichannel pipette to prevent evaporation.
17. Incubate the plate in a humidified, regular oxygen incubator at 37°C and 5% CO_2 until weekly media refreshments starting from week 1.

Week 1 and 2: refresh media to support neuronal maturation

1. Prepare *Co-culture medium without doxycyclin*: Neurobasal medium + 10 ng/mL NT3 + 10 ng/mL BDNF. 100 µL per well/3D culture, prewarmed in waterbath
2. Aspirate 90 µL of the spent medium and gently add 100 µL fresh *Co-culture medium without doxycyclin* per well with a P200 or multichannel pipette, but prevent touching the 3D cell suspension.
    - Note: Tilting the plate and aspirating the medium from the well rim helps to prevent touching the 3D cell suspension.
3. Incubate the plate in a humidified, regular oxygen incubator at 37°C and 5% CO_2.

Week 3: refresh media to support final neuronal maturation

1. Prepare *Co-culture medium without doxycyclin*: Neurobasal medium + 10 ng/mL NT3 + 10 ng/mL BDNF. 180 µL per well/3D culture, prewarmed in waterbath
2. Aspirate 90 µL of the spent medium and gently add 180 µL fresh *Co-culture medium without doxycyclin* per well with a P200 or multichannel pipette, but prevent touching the 3D cell suspension.
   - ➢ Note: Tilting the plate and aspirating the medium from the well rim helps to prevent touching the 3D cell suspension.
   - ➢ Note: Compared to week 1 and 2, a larger volume of fresh medium is used since we observed higher medium expenditure from this timepoint.
3. Incubate the plate in a humidified, regular oxygen incubator at 37°C and 5% CO_2 until fixation at week 4.
